# Long-term follow-up study on obstructive hypertrophic cardiomyopathy patients treated with disopyramide: evidences of a notable trend in symptom control within a real-world clinical setting

**DOI:** 10.3389/fcvm.2024.1416600

**Published:** 2024-08-14

**Authors:** Gaetano Todde, Lorenzo Lupo Dei, Roberto Polizzi, Domenico Gabrielli, Grazia Canciello, Silvio Romano, Felice Borrelli, Geza Halasz, Leopoldo Ordine, Salvatore Di Napoli, Daniela Pacella, Raffaella Lombardi, Giovanni Esposito, Federica Re, Maria-Angela Losi

**Affiliations:** ^1^ Department of Advanced Biomedical Sciences, University Federico II, Naples, Italy; ^2^ Department of Advanced Biomedical Sciences, San Camillo Hospital, University Federico II Naples, Rome, Italy; ^3^ Cardiology, Department of Life, Health and Environmental Sciences, University of L'Aquila, L'Aquila, Italy; ^4^ Department of Public Health, University Federico II, Naples, Italy

**Keywords:** hypertrophic cardiomyopathy, disopyramide, obstruction, therapy, symptoms

## Abstract

**Background:**

In obstructive hypertrophic cardiomyopathy (HOCM), disopyramide is used in patients who remain symptomatic despite *β*-blockers or verapamil. However, effectiveness of disopyramide therapy has not been clearly established due to inconsistent definition of responders and the insufficient length of follow-ups reported in literature. To address these shortcomings, we have conducted a retrospective analysis from detailed databases with long follow-up, from two HCM Referral Centers.

**Methods:**

62 symptomatic HOCM patients (43% women, age 52 ± 14 years) with left ventricular (LV) outflow tract gradient (LVOTG) ≥ 50 mmHg at rest or during provocation, were recruited from two Italian Centers. Disopyramide was added as second-line therapy in the patients in whom symptoms persisted despite classic pharmacologic treatment. Patients in NYHA class > II at baseline who reached NYHA class II or I, and patients in NYHA class II at baseline who reached NYHA class I or symptoms stabilization were defined as responders.

**Results:**

At follow-up, (mean 4.4 years, IQR 1.1–6.6 years), 47 patients (76%) were responders, whereas 15 (24%) were no-responders. Responders showed larger LV diastolic volume index (LVEDVi) at baseline as compared to no-responders (61 ± 14 vs. 49 ± 16 ml, respectively, *p* = 0.018), and, at follow-up, reached lower LVOTG than no-responders (43 ± 32 vs. 66 ± 28 mmHg, respectively, *p* = 0.013), with a LVOTG <50 mmHg more represented in responders than in no-responders (75% vs. 25%, respectively; *p* = 0.004). No side effects requiring discontinuation of the therapy were recorded.

**Conclusion:**

HOCM patients treated with disopyramide as second-line therapy in a quite long-follow-up showed a significant improvement of symptoms, which avoided SRT in up to 70% of them. Moreover, our data suggest that a larger LVEDVi at baseline identify the subgroup of patients who benefit the most from the therapy in terms of symptoms and reduction of LVOTG below 50 mmHg during treatment. We will discuss specific situations where disopyramide may be preferred over myosin inhibition to ensure that effective therapeutic options are fully considered and not prematurely dismissed.

## Introduction

Hypertrophic cardiomyopathy (HCM) is clinically defined by an increased thickness of left ventricular (LV) walls that cannot be solely attributed to abnormal loading conditions such as hypertension or valve disease (1, 2).

The disease's pathophysiology is characterized by diastolic dysfunction and LV outflow tract gradient (LVOTG) (3). LVOTG is typically dynamic and if ≥30 mmHg has been shown to be a strong, independent predictor of progression to severe heart failure symptoms and increased mortality (4).

Current guidelines recommend treating patients with obstructive HCM (HOCM) in presence of symptoms, along with a LVOTG ≥50 mmHg at rest or during provokable maneuvers (1). The initial approach involves, in Class I, pharmacological treatment with non-vasodilating *β*-blockers or non-dihydropyridine calcium channel blockers, whereas as second-line therapies (5) disopyramide or mavacamten, can be employed (6–16). If patients remain severely symptomatic, i.e., in NYHA class III or IV despite optimized medical therapy, invasive treatment to reduce obstruction is indicated.

Patients with persistent New York Heart Association (NYHA) class III or IV and LVOTG ≥50 mmHg are considered not responders to medical therapy (1). This approach is based on the assumption that symptoms are primarily due to LVOTG. However, it's important to note that responders are commonly but inaccurately identified as those who exhibit improvements in both NYHA class and LVOTG. It's crucial to understand that LVOTG reduction alone doesn't necessarily warrant subsequent invasive treatments. The decision for further interventions, such as septal reduction therapy (SRT), varies significantly across studies, with indications ranging from one-third to nearly half of the patient population studied. This lack of consistency underscores the complexity of managing HCM and the need for individualized treatment approaches based on comprehensive clinical assessment rather than relying solely on LVOTG reduction as a treatment endpoint (17, 18). In addition, although Sherrid et al. demonstrated the efficacy and safety of disopyramide in the treatment of symptomatic HOCM almost 20 years ago, subsequent studies, even performed in the real world, have been conducted only in short follow-up (19). Hence, the purpose of our study is to evaluate the efficacy of the treatment with disopyramide on symptoms, independently of the degree of LVOTG. For this purpose, we conducted a retrospective analysis including HOCM patients from two HCM centers over a long follow-up period defining as responders, the patients in which the treatment ameliorated or stabilized the symptoms to NYHA class II.

## Methods

### Population

**Figure 1:**
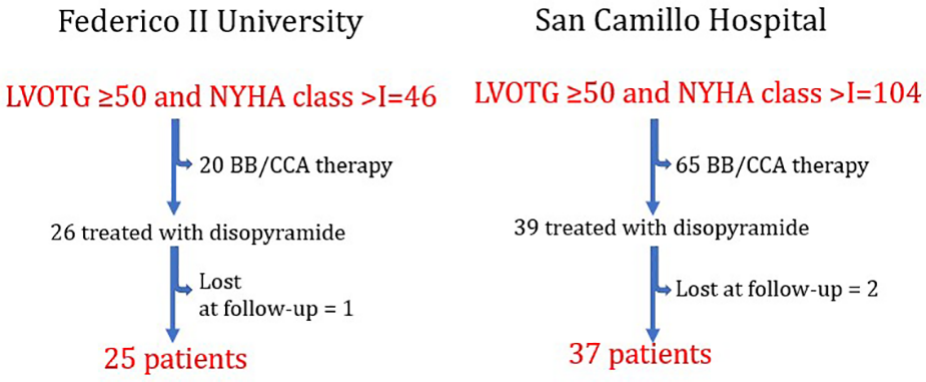
Chart illustrating the criteria used to identify the studied population, divided basing on afferent institution.

150 symptomatic HOCM patients from the HCM cohort of the Federico II University of Naples and of San Camillo-Forlanini Hospital in Italy, were considered for the study. All enrolled patients gave informed consent to participate in the study. The diagnosis of HCM was made by the two-dimensional echocardiographic evidence of LV maximal wall thickness (MWT) ≥ 15 mm in the absence of other cardiac or systemic cause capable of inducing a similar magnitude of left ventricular hypertrophy (20–22). For the present analysis, we excluded patients who had already undergone SRT. In addition, we considered only patients with maximal tolerated therapy, so only those in disopyramide, as second-line therapy. Figure 1 reports the exclusion criteria used for the analysis. The final population was of 62 HCM patients (43% women, age 52 ± 14 years).

### Definitions

Patients were defined: (1) asymptomatic, NYHA Class I, if the patient reported no shortness of breath limiting ordinary physical activity; (2) symptomatic, NYHA class II, if the patient reported mild shortness of breath and slight limitation during ordinary activity; (3) symptomatic, NYHA class III, if marked limitation in activity due to symptoms, even during less-than-ordinary activity were reported or if syncope, diagnosed as related to LVOTG occurred, NYHA class IV, if the patient was unable to carry on any physical activity without discomfort. In our clinics, we treat with medication only those patients who have obstruction and are at least in NYHA class II.

The presence of obstruction alone, as reported by the guidelines, is not an indication for treatment. Therefore, if during treatment the symptoms disappeared, meaning the patient moved to NYHA class I, they were considered responders. At the same time, patients with HOCM who, during follow-up, did not have symptoms severe enough to warrant invasive interventions—patients who moved from NYHA class III to class II, or those who remained in class II without symptom worsening—were also considered responders. Thus, patients were divided in: (1) Responders, those patients in NYHA III at baseline, who reached NYHA II or I, or those in NYHA class II at baseline who reached NYHA class I or showed stabilization of symptoms; (2) no-responders: patients who did not show any of the previous conditions, i.e., in NYHA class III at baseline that did not change during follow-up, or patients whose NYHA progressed to class III during follow-up or patients showing syncope diagnosed to be related to LVOTG.

LVOTG was defined by echocardiographic evidence of a peak instantaneous Doppler LVOTG ≥50 mmHg at rest or during physiological provocations such as Valsalva maneuver, standing or exercise. Care was taken to avoid contamination with the mitral regurgitation jet, which might cause an overestimation of the obstruction (23).

### Treatment and follow-up

Non-vasodilating beta-blockers or non- dihydropyridine calcium channel blockers, titrated to maximum tolerated dose, were used as first-line therapy in patients with symptomatic HOCM in both Institution. Disopyramide was routinely initiated as second-line therapy in addition to a beta-blocker or, if this was not possible, with verapamil or diltiazem to control symptoms. The exclusion criteria for initiating disopyramide were a baseline QTc > 500 msec, LV ejection fraction <50%, advanced A-V blocks or bi-fascicular block, concurrent administration of other antiarrhythmics.

A clinical follow-up with cardiac examination, ECG and echocardiogram every 1/2 weeks since the initiation of disopyramide was performed. The initial dosage of disopyramide was of 221 ± 33 mg daily. If symptoms did not improve, the dose was increased by 100 mg/day, every 1/2 weeks, up to a maximum tolerated dose of 500 mg/day. Once a clinical response to therapy was obtained, future follow-ups were scheduled every 6 months.

During follow-up, the dose of disopyramide was reduced or discontinued in case of anticholinergic symptoms, QTc-interval prolongation >550 ms or LV systolic disfunction (LV Ejection fraction <50%).

### Outcome

The primary endpoint was considered the patients clinical response to disopyramide as reported above, i.e., responders and no-responders. The end of the follow-up was the date of last visit for responders, and the date of the visit that referred the patient for SRT in no-responders.

### Statistics

Normally distributed continuous variables are described as mean ± standard deviation. Categorical variables are described as number (percentage). Unpaired *T*-test was used to compare baseline characteristics of responders and no-responders patients. ANOVA was used to test differences between responders and no-responders. Multivariate analysis was performed to identify predictors of responders to disopyramide. A two-tailed *p*-value < 0.05 was considered statistically signiﬁcant in all analyses. Data were analyzed using SPSS (version 26.0; SPSS, Chicago, IL) and R Statistical Software version 4.3.0.

## Results

**Figure 2:**
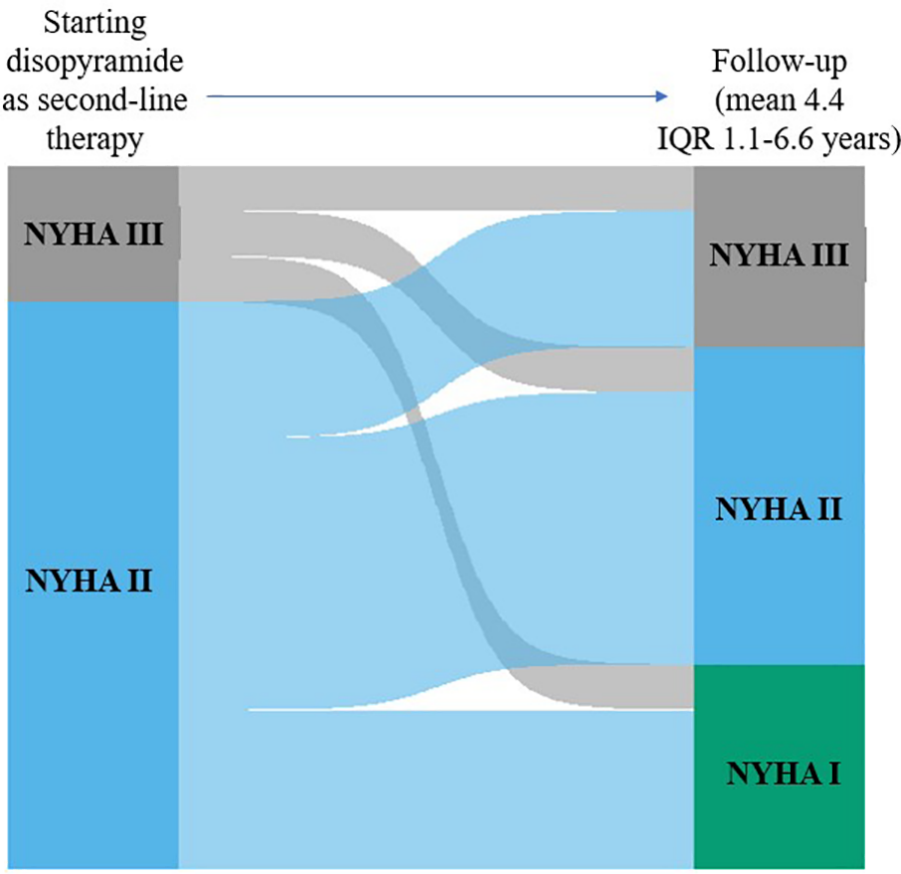
Individual changes in NYHA functional class from baseline to early follow-up and to early follow-up to late follow-up. The height of each bar is proportional to the number of patients with the corresponding NYHA functional class, and the width of the ends of each flow line is proportional to the number with the given pattern of change of NYHA functional class.

The mean dose of disopyramide during follow-up was of 349 ± 27 mg/day. At follow-up, (4.4 ± 3.6 years, 0.4 to 14 years IQR 1.1–6.6 years), 47 patients (76%) were responders, whereas 15(24%) patients were no-responders, and thus referred to SRT The individual changes in NYHA functional class are reported in Figure 2. Table 1 reports baseline differences between responders and no-responders. Most clinical and echocardiographic data were not different between responders and non-responders. However, LV end-diastolic volume index was higher at baseline in responders than in no-responders (61 ± 14 vs. 49 ± 16 ml/m^2^, respectively, *p* = 0.018). There were no differences at baseline of LVOTG between responders and no-responders (Table 1), whereas at follow-up responders showed a significant lower LVOTG than no-responders (43 ± 32 vs. 66 ± 28 mmHg, respectively, *p* = 0.013), with LVOTG <50 mmHg more represented in responders than in no-responders (75% vs. 25%, respectively; *p* = 0.004). Multiple regression analysis conducted including the variable significantly different between responders and no-responders, i.e., LV end diastolic volume index at baseline and LVOTG at follow-up, finds that LV end-diastolic volume index was predictor of responders (OR 1.060; 95% Clo 1.008–1.115; *p* = 0.022).

**Table 1:** Baseline characteristic of the studied population divided basing on clinical response to disopyramide at follow-up.

Variable	Responders (47)	No-Responders (15)	*p*
Age (years)	52 ± 15	50 ± 13	0.830
Female sex (#, %)	19, 56	16, 57	0.791
BSA (kg/m2)	1.8 ± 0.4	1.8 ± 0.4	0.836
NYHA class > II (#, %)	8, 17	4, 27	0.410
Heart rate (bpm)	65 ± 13	64 ± 11	0.830
Left atrial diameter (mm)	47 ± 9	47 ± 9	0.375
LV maximal wall thickness (mm)	22 ± 5	23 ± 5	0.353
Left atrial volume index(ml/m^2^)	46 ± 16	41 ± 14	0.304
Moderate to severe mitral regurgitation (#, %)	21, 46	7, 47	0.945
LV end-diastolic volume index (ml/m^2^)	61 ± 14	49 ± 16	0.018
LV ejection fraction (%)	71 ± 7	72 ± 6	0.740
LV outflow tract obstruction (mmHg)	78 ± 25	79 ± 38	0.857
E/A	1.4 ± 1.2	2.2 ± 2.9	0.370
Beta-blockers (%)	41, 87	11, 73	0.203
Non-dihydropyridine calcium channel blockers (%)	6, 13	4, 27	0.304
Disopyramide dosage (mg/daily)	220 ± 29	223 ± 46	0.775

Adverse reactions to disopyramide occurred in 6 patients during follow-up. Among these patients, anticholinergic symptoms were observed in 2 patients, while QTc prolongation was observed in 4 patients. In all 6 cases the discontinuation of the medication was not necessary as a reduction of the dosage was sufficient to reverse the negative effects.

## Discussion

To the best of our knowledge, this study represents the first comprehensive examination of the effects of disopyramide in symptomatic HOCM patients, conducted in a real-world scenario encompassing a relatively lengthy follow-up period, at least from Europe. Our findings reveal that over a mean follow-up of 4.4 years, disopyramide treatment successfully prevented the need for invasive SRT in up to 70% of patients in two tertiary centers. This result holds particular significance given that individuals with HOCM are typically not elderly, suggesting the necessity for prolonged treatment, as confirmed in our study, where 50% of patients were younger than 50 years, and only 26% were older than 60 years.

It is noteworthy that while disopyramide underwent testing in a multicenter study almost 20 years ago (19), with subsequent larger studies confirming the safety of the drug (24), since then few investigations have explored the long-term effects of this medication. The dearth of extensive research in real-world clinical settings on disopyramide may stem from physicians' hesitancy, with many being reluctant to prescribe class I anti-arrhythmic agents for structural heart diseases This attitude persists despite the fact that disopyramide has proven to inhibit multiple ion channels, leading to lower Ca transients and force, and shortens action potentials, thus reducing cellular arrhythmias (25). This electrophysiological profile of disopyramide explains the efficient reduction of outflow gradients but also the limited prolongation of the QT interval and the absence of arrhythmic side effects observed (25). Time will tell if significant challenges arise with the use of newer agents such as mavacamten, a myosin inhibitor.

Recently, in Italy mavacamten has entered the “compassionate” use phase. This means that the drug is already available and can be supplied by the manufacturer for patients with HOCM, upon request by expert cardiologists. However, at moment initiation requires patient genotyping for Cytochrome P450 2C19 to determine the appropriate dosage which could be not available in some centers, and has not been required by the U.S. FDA.

Additionally, patients on this treatment must undergo many echocardiograms within 12 weeks to monitor potential reductions in LV ejection fraction (26–28). Hence, it is imperative to conduct studies aimed at discerning potential differences in physician perceptions between older and newer drugs. Unfortunately, trials on disopyramide are lacking, and current attention has shifted towards novel medications like myosin inhibitors, for which long-term follow-up data is currently unavailable. Efforts should be directed towards bridging this gap in understanding between established and emerging treatments for HOCM. Furthermore, it is noteworthy that disopyramide was not discontinued in any of our patients, with a reduction in dosage only recommended for a select few. This outcome underscores the safety of the treatment. If substantiated by further studies, this finding will necessitate future comparisons with the safety of myosin inhibitors. which have been withdrawn in up to 5% of patients due to a significant reduction in LV ejection fraction (26–28).

The average dose of disopyramide in our study was around 340 mg/day, which is considered a low dose. Higher dosages have been shown to yield better results in reducing symptoms and gradient, with a mean effective dose of 501 mg/day (29). Conversely, the high referral rate for SRT reported in a recent study, may be attributed to the low dose of medication used (30). Though the exact dosage was not specifically detailed by the authors, it seems that most patients received 250 mg/day, thus explaining their failure to achieve symptom relief (30). Therefore, our results, in conjunction with other studies, indicate a dose-dependent response to disopyramide treatment (31).

The positive outcomes we found in our study also provide insights into how responders should be defined in clinical practice. A clinical response to treatment in patients with symptomatic HOCM should be based on an improvement or stabilization of NYHA class to at least NYHA II, unless severe symptoms other than dyspnea, such as syncope related to LVOTG, are diagnosed. Our approach aligns with current guidelines recommending treatment for patients with HOCM in the presence of NYHA class > II and a LVOTG >50 mmHg at rest or during provokable maneuvers. This perspective reflects the idea that symptoms are primarily attributed to LVOTG if confirmed over time.

Due to this approach, responders are occasionally misidentified as those showing a reduction in both NYHA class and LVOTG, even though LVOTG *per se* is not an indication for subsequent invasive treatment. In a recent paper examining the effects of treatment in a real clinical setting over a short follow-up of 12 months, 45% of HOCM patients treated with disopyramide were referred for Septal Reduction Therapy (SRT) (30). This high referral rate, in addition to the low dosage of disopyramide reported above, may be attributed to the definition of responders, characterized by achieving a functional class NYHA I and an LVOTG <30 mmHg—a condition never considered as a primary endpoint in studies assessing the effects of disopyramide or myosin inhibitors (19, 24, 26–28).

Based on our findings, individuals who responded positively to the therapy were more likely to exhibit a larger baseline LV end-diastolic volume compared to non-responders. It is known that patients with obstruction generally demonstrated smaller LV end-diastolic diameters than those without (3). This observation underscores that smaller cavities are more susceptible to developing obstruction. Moreover, smaller cavities have been associated with functional limitations, independent of the presence of obstruction. This is likely due to the restrictive pathophysiology induced by small cavities (32). Considering this collective evidence, we can hypothesize that larger cavities are more inclined to alleviate obstruction during treatment and are more likely to improve their filling in the presence of reduced obstruction, and consequently symptoms. Habib et al, found that a lower ejection fraction was the best predictor of a poor response to disopyramide (33). In fact, in half of their no-responders LV ejection fraction was lower than 60%; in our report, however, LV ejection fraction was not different at baseline between responders and no-responders, with only 2 patients showing LV ejection fraction <60%. Several factors make our study and Habib's study not directly comparable: Habib et al. defined responders as patients who experienced a reduction of at least 30% in LVOTG from baseline. This definition does not align with any established criteria in the literature, which complicates direct comparisons. It is likely that Habib et al. treated patients with both disopyramide and low ejection fraction. In such patients, the expected effect of disopyramide (i.e., reduction in ejection fraction) was already present at baseline. As a result, disopyramide may not have been effective in further reducing LVOTG in these patients.

It is not entirely clear why a reduction rather than an elimination of LVOTG leads to an improvement in symptoms. In the study by Sharma et al. in a large HCM population, the percent-predicted peak VO2 was similar in patients with and without resting LVOTG, although there was an inverse correlation between the magnitude of LVOTG and peak VO2 suggesting that the degree rather than its presence has adverse pathophysiologic consequences (34). In a recent paper from part of the authors of the present paper, among 22 HOCM patients treated for 3 months with disopyramide, peak VO2 decreased, probably because heart rate was blunted during exercise, however with the patients filling better, as demonstrated by the trend of NYHA class and of Quality-of-Life score (35).

Overall, these observations confirm that the pathophysiology of HCM is intricately linked not only to obstruction but also to a complex interplay among microcirculation, ischemia, fibrosis, hypertrophy, and diastolic dysfunction (36). In particular, the uncoupling of gradient reduction and symptom improvement might be attributed to an enhancement in diastolic function resulting from a significant decrease in cytosolic calcium levels by disopyramide, as demonstrated by Coppini et al. (25). The relationship between cytosolic calcium and diastolic dysfunction is well documented. Louch et al. (37) discuss how diastolic calcium homeostasis plays a critical role in both normal and failing myocardium, highlighting that disturbances in calcium regulation can lead to diastolic dysfunction. Moreover, diastolic tone, while essential for cardiac performance, can become detrimental when dysregulated, further emphasizing the importance of proper calcium handling in maintaining diastolic function (38).

These insights collectively suggest that improvements in diastolic function via cytosolic calcium reduction could potentially explain the observed discrepancies between gradient reduction and symptom relief in some HOCM patients.

Our results could underscore the necessity of conducting comparative studies between disopyramide and newer drugs before considering its abandonment. However, it is unlikely that such studies will be conducted because myosin inhibitors are significantly stronger negative inotropes, more effective at relieving symptoms, free from electrophysiologic side effects, and more precisely targeted at the sarcomere (26–28). Additionally, funding for such studies is improbable.

Despite this, there are specific situations where disopyramide may still play an important role due to its unique pharmacokinetic, pharmacologic, and cost characteristics. Disopyramide can be preferred when immediate gradient reduction is needed in cases of severe heart failure due to obstruction or hypotension, as myosin inhibitors take days to weeks to reach steady-state concentrations. Disopyramide, when administered in adequate doses, has an immediate effect from the first dose (39). This rapid action is particularly important *in situ*ations of hemodynamic compromise following non-cardiac surgery or during concurrent severe non-cardiac illness.

Furthermore, disopyramide possesses antiarrhythmic properties, which myosin inhibitors lack, making it a valuable option for patients with both HOCM and atrial fibrillation. Lastly, the high cost of myosin inhibitors may render them inaccessible to some patients, whereas disopyramide remains a viable and more affordable pharmacologic treatment option.

### Strengths and limitations

The strength of our paper is the very long-follow-up in a clinical real scenario. The main limitations of our study are the small cohort of patients under examination and the retrospective nature of the study. Another limitation of our study is the method used to assess exercise tolerance, which was done through the NYHA classification. This assessment relies in part on the physician's interpretation of the symptoms described by the patient. However, it is important to note that clinical guidelines and routine practice in outpatient settings are based on this type of assessment.

## Conclusions

Treatment of HOCM should not only directed to reduction of LVOTG, in that gradient improvement is not the only arbiter of success, but firstly to improvement of symptoms. In this contest, we can conclude from our study that disopyramide represents a highly effective drug in a quite long term, with a good clinical profile capable of avoiding the use of SRT in up to 70% of patients. It seems that the response to therapy is not only related to the reduction of LVOTG, and, finally, the patients who might benefit the most from the therapy are those with larger LV diastolic volumes. Our results highlight the need for comparative studies between disopyramide and newer drugs, although such studies are unlikely due to the superior efficacy, specificity, and fewer side effects of myosin inhibitors, as well as funding challenges. Despite this, disopyramide remains crucial for immediate gradient reduction in severe heart failure cases and is a cost-effective option with antiarrhythmic benefits, making it suitable for patients with HOCM and atrial fibrillation.

## Data Availability

The raw data supporting the conclusions of this article will be made available by the authors, without undue reservation.
